# In situ Electrochemical-AFM Study of LiFePO_4_ Thin Film in Aqueous Electrolyte

**DOI:** 10.1186/s11671-016-1446-1

**Published:** 2016-04-27

**Authors:** Jiaxiong Wu, Wei Cai, Guangyi Shang

**Affiliations:** Department of Applied Physics, Beihang University, Beijing, 100191 People’s Republic of China; Key Laboratory of Micro-nano Measurement-Manipulation and Physics (Ministry of Education), Beihang University, Beijing, 100191 People’s Republic of China

**Keywords:** LiFePO_4_ thin film, Radio frequency magnetron sputtering, In situ electrochemical-AFM

## Abstract

Lithium-ion (Li-ion) batteries have been widely used in various kinds of electronic devices in our daily life. The use of aqueous electrolyte in Li-ion battery would be an alternative way to develop low cost and environmentally friendly batteries. In this paper, the lithium iron phosphate (LiFePO_4_) thin film cathode for the aqueous rechargeable Li-ion battery is prepared by radio frequency magnetron sputtering deposition method. The XRD, SEM, and AFM results show that the film is composed of LiFePO_4_ grains with olivine structure and the average size of 100 nm. Charge-discharge measurements at current density of 10 μAh cm^−2^ between 0 and 1 V show that the LiFePO_4_ thin film electrode is able to deliver an initial discharge capacity of 113 mAh g^−1^. Specially, the morphological changes of the LiFePO_4_ film electrode during charge and discharge processes were investigated in aqueous environment by in situ EC-AFM, which is combined AFM with chronopotentiometry method. The changes in grain area are measured, and the results show that the size of the grains decreases and increases during the charge and discharge, respectively; the relevant mechanism is discussed.

## Background

With the wide utilization of portable electrical devices and the growing demand for new energy, the development of high-performance and environmentally friendly energy storage devices is becoming increasingly important. Among the kinds of energy storage devices, lithium-ion (Li-ion) batteries are known to be the most promising medium for energy storage and they have been widely used in various kinds of electronic devices in our daily life [1]. In Li-ion battery components, the cathode material plays a very important role since it determines the performance of the Li-ion battery. Lithium transition metal oxides, such as LiMn_2_O_4_, LiCoO_2_, and LiNiPO_4_, have been widely used as cathode materials of Li-ion batteries. Among these, lithium iron phosphate (LiFePO_4_), firstly introduced as a cathode by Padhi [2], has been a promising cathode material because of its high theoretical specific capacity (170 mAh g^−1^) and thermal stability at high temperature [3].

As known to all, organic liquids have been widely used as the electrolyte in conventional Li-ion batteries. However, major drawbacks of the organic electrolyte are obvious such as (a) when the battery is discarded, the electrolyte may leak into the land and cause environmental problems [4, 5]; and (b) the electrolyte makes the manufacture process of the battery more complicated and expensive. For these reasons, the use of aqueous electrolyte in Li-ion battery would be an alternative way to develop low cost and environmentally friendly batteries [6]. Fortunately, the electrochemical performance of LiFePO_4_ as a cathode in aqueous rechargeable Li-ion battery (ARLB) has been studied. It was found that LiFePO_4_ electrode could undergo lithium-ion extraction and intercalation process in Li_2_SO_4_ electrolyte at the safe potential window without the electrolysis of water in electrolyte [7]. The electrochemical cell in aqueous electrolyte was charged and discharged at a current density of 20 μA cm^−2^ and exhibited an initial discharge capacity of 109 mAh g^−1^ [8]. The main problem is that the cycling capacity of LiFePO_4_ cathode suffers a big decrease and its capacity loss rate in aqueous electrolyte is much faster than that in organic electrolyte. It has been reported that in aqueous electrolyte, the estimated average discharge capacity for 20 cycles is about 29 μAh cm^−2^, while it is 36 μAh cm^−2^ in nonaqueous electrolyte [9, 10]. However, the mechanism or reason for the capacity degradation is not completely understood. In order to improve the cycling performance, therefore, it is important to study the aging characteristics of ARLB. One particular aging-dependent and compelling phenomenon is the marked volume change of the cathode material due to insertion and de-insertion of lithium ions.

A lot of research work on the aging phenomenon of cathode and anode in Li-ion batteries has been performed by using various in situ and ex situ techniques, such as scanning electron microscopy [11, 12], transmission electron microscopy [13, 14], X-ray photoelectron spectroscopy [15, 16], Raman spectroscopy [17, 18], and atomic force microscopy (AFM). Among these techniques, electrochemical AFM (EC-AFM), combining AFM with electrochemical method, is a powerful in situ tool to study both surface morphology and chemistry of the Li-ion battery electrodes during the cycling of an electrochemical cell. However, the sample used for EC-AFM to study the morphology change and aging phenomenon in some researches was extracted from commercially available battery [19, 20]. These samples resulted in different potential distributions and uncertain electrode surface since the sample generally contains active materials, organic binders, and conductive additives.

In this paper, we focus on in situ EC-AFM characterization of LiFePO_4_ thin film as the cathode of Li-ion battery in aqueous electrolyte. Nanoparticle powder was synthesized through the solvothermal method, and thin film electrodes were then prepared by radio frequency magnetron sputtering. The electrochemical performance of LiFePO_4_ films was measured in 1 M Li_2_SO_4_ electrolyte by the electrochemical workstation, and capacity was also tested. A special homemade electrochemical cell was designed for in situ EC-AFM characterization. The surface morphology changes of LiFePO_4_ thin film electrodes were investigated by the in situ EC-AFM system. The changes in the grain size of the LiFePO_4_ film during charge and discharge processes were observed and discussed.

## Methods

The LiFePO_4_ powder was synthesized by a solvothermal method with stoichiometric amounts of LiOH·H_2_O, FeSO_4_·7H_2_O, and H_3_PO_4_ in a molar ratio of 3:1:1, and ethylene glycol was applied as the solvent. Detailed procedures are described as follows: two solutions of ferrous sulfate and lithium hydroxide were made by dissolving FeSO_4_·7H_2_O and LiOH·H_2_O in ethylene glycol, respectively. Then, 0.1 M H_3_PO_4_ was mixed with 0.3 M LiOH·H_2_O solutions under sufficiently mechanical stirring. After that, 0.1 M FeSO_4_·7H_2_O solutions were slowly added under stirring, resulting in a green-colored suspension. The solution was transferred into a Teflon-lined stainless steel autoclave. The autoclave was sealed and set at 180 °C and then cooled down after 9 h. The products were washed with deionized water and ethanol three times and then dried under vacuum at 80 °C for 12 h. The LiFePO_4_ powder was then cold pressed and sintered at 750 °C under Ar/H_2_ (2 % H_2_) atmosphere for 24 h. The size of the target is 50 mm in diameter and 3 mm in thickness.

LiFePO_4_ film was deposited on a Au/Si substrate by radio frequency magnetron sputtering with the target. The Au film was pre-deposited on a Si substrate by radio frequency magnetron sputtering in Ar (purity 99.99 %) for 90 s as the current collector. The sputtering process of the LiFePO_4_ target was performed following Ar ambient with a pressure of 2.7 Pa and flow rate of 50 standard cubic centimeters per minute (sccm). The distance between the target and the substrate was 5 cm, and a magnetron sputtering power of 70 W was applied to the target. In order to eliminate contaminants on the surface of the target and substrate, it is necessary to pre-sputter under identical conditions before the sputtering of the LiFePO_4_ thin film. During deposition process, the temperature of the substrate was 300 °C. Finally, the film was annealed under vacuum at 500 °C for 4 h.

The composition and crystal structure of powder and thin film were characterized by X-ray diffraction with Ni-filtered Cu Kα radiation operated at 40 kV and 30 mA (Shimadzu, XRD-6000). Field-emission scanning electron microscopy (Hitachi S-4800), operated at an accelerating voltage of 20 kV, was utilized to determine the grain morphology and size. Atomic force microscope (CSPM5500, Benyuan Co. China) was used to characterize the surface of the film electrode under atmosphere and aqueous environments.

The electrochemical properties of the LiFePO_4_ powder and film electrodes were characterized by a standard three-electrode system. The LiFePO_4_ powder electrode was prepared as follows: LiFePO_4_ powder was mixed with polyvinylidene fluoride (PVDF) and acetylene black in a weight ratio of 8:1:1. Then, the mixed powder was dissolved in *N*-methylpyrrolidone (NMP), and the suspension was stirred vigorously for 1 h. Subsequently, the prepared suspension was uniformly coated on platinum foil and dried at 80 °C for 6 h under vacuum environment. Finally, the LiFePO_4_ cathode was formed and served as a working electrode. The cyclic voltammetry and chronopotentiometry (CP) measurements were performed in 1 M Li_2_SO_4_ aqueous solution at room temperature. A platinum plate and saturated calomel electrode (SCE, *E* = 0.245 V vs. standard hydrogen electrode, NHE) were employed as counter electrode and reference electrode, respectively. The measurements were collected at a scan rate of 1 mV s^−1^ in a range of −0.8 to 1.2 V safe voltage window. All potentials were measured with respect to saturated calomel electrode.

Charge-discharge measurement of the thin film battery in this research was conducted by Swagelok cell. The deposited LiFePO_4_ thin film was employed as the positive electrode, and the Pt foil served as the negative electrode. The electrolyte was 1 M Li_2_SO_4_ aqueous solution. Galvanostatic charge-discharge tests were carried out by the Neware Battery Test Station in voltage range from 0 to 1 V (vs. SCE) at the constant current of 10 μAh cm^−2^ at room temperature.

**Fig. 1:**
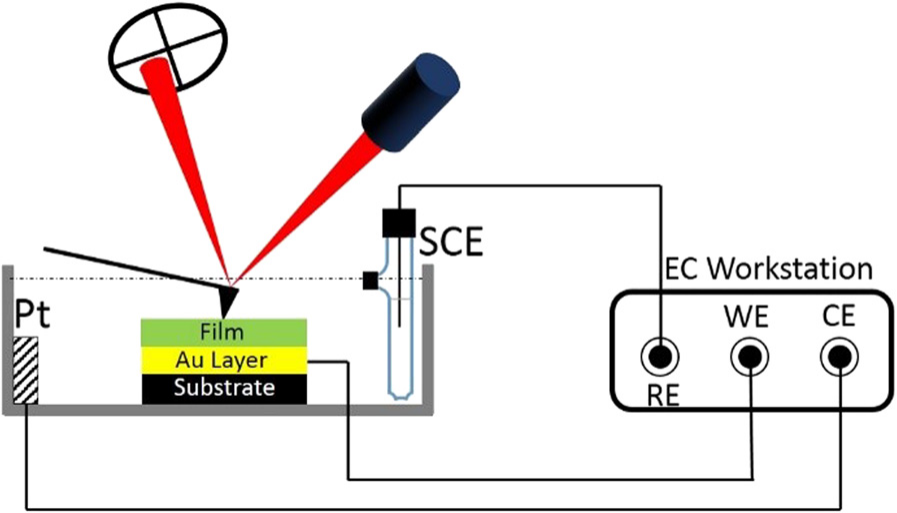
Schematic of the homemade in situ electrochemical-AFM measurement system, where CE, WE, and RE represent counter electrode, working electrode, and reference electrode, respectively

In situ EC-AFM measurement was performed by AFM system (CSPM-5000, Benyuan Co.) combined with the CP measurement in electrochemical workstation (CHI600D) in a special home-designed liquid cell, as shown in Fig. 1. The outside diameter of the electrochemical cell is 30 mm, the inner diameter is 20 mm, and the depth is 5 mm. The thin film electrode was placed at the bottom of the cell. The platinum foil that is used as counter electrode was placed along the cell wall and saturated calomel electrode was fixed beside the thin film. Three electrodes were well electrically connected to the electrochemical workstation. After three electrodes were fixed well, 1 M Li_2_SO_4_ aqueous solution was dropped into the cell and the film electrode was totally immerged into the solution. AFM images were taken at different time periods as follows: (a) before the cell was charged, (b) after the cell was charged by applying a positive voltage, and (c) after the cell was discharged by applying a negative voltage, respectively. AFM imaging was carried out in contact mode using silicon nitride probes with a force constant of 0.2 N m^−1^ and a resonant frequency of 13 kHz. Electrochemical experiments were performed with chronopotentiometry by the CHI600D workstation. AFM images were processed using Image J Software. All measurements were carried out at room temperature.

## Results and Discussion

**Fig. 2:**
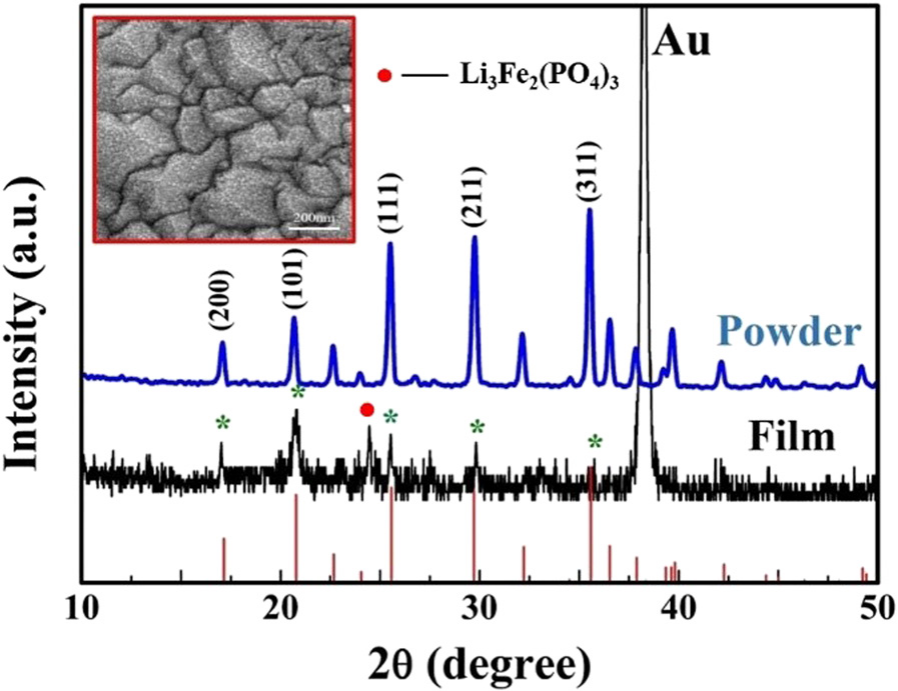
XRD patterns of the LiFePO_4_ powder and the film, as well as SEM photograph of the thin film

The XRD pattern of the thin film deposited on Au/Si substrate is shown in Fig. 2, and the XRD pattern of the LiFePO_4_ powder scraped from the target is also shown for comparison. These results indicate that the powder exhibits crystallinity of an olivine structure and the orthorhombic system with pnmb space group (JCDPS Card No.81-1173). In spite of the strong peak from the Au substrate, the thin film also shows crystallinity and LiFePO_4_ predominant phase with a relatively strong peak of (101) predominant orientation. It should be noticed that several peaks of the LiFePO_4_ thin film including (200), (101), (111), (211), and (311) are able to be identified, which are correspond to diffraction peaks of the LiFePO_4_ powder, implying that the film also has the olivine structure. Some of the peaks overlap with the peaks from the Au film pre-deposited on Si substrate, and the intensity ratios of peaks are slightly different from the powder target. However, small amount of Li_3_Fe_2_(PO_4_)_3_ impurity phase is showed in the peak at 23.8°, which is consistent with JCDPS Card No.78-1106 of Li_3_Fe_2_(PO_4_)_3_. The formation of Li_3_Fe_2_(PO_4_)_3_ may result from the reaction with the residual O_2_ and H_2_O vapor from the chamber [21] because the target made by the powder have no impurity phase as shown in Fig. 2. Nevertheless, the main X-ray diffraction features of the thin film are consistent with olivine LiFePO_4_ phase. Moreover, the morphology of the thin film was characterized by FE-SEM and the result is given in Fig. 2, which shows that the film surface is composed of densely packed nano-grains with the size of ~100 nm and distinct boundaries.

**Fig. 3:**
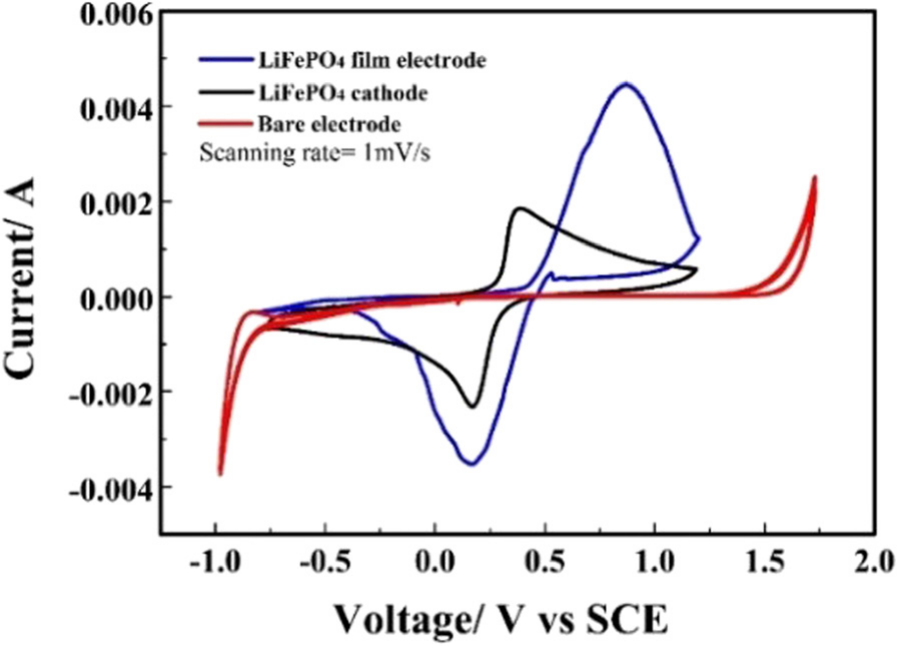
Cyclic voltammetry curves of the LiFePO_4_ powder and the LiFePO_4_ thin film in 1 M Li_2_SO_4_ aqueous electrolyte

The electrochemical performance of the LiFePO_4_ powder and the film were characterized by cyclic voltammetry with a three-electrode system in 1 M Li_2_SO_4_ aqueous electrolyte. The cyclic voltammetry experiments were performed in the range of −0.8 to 1.2 V at a scan rate of 1 mV s^−1^, and results were shown in Fig. 3. As seen from Fig. 3, both the powder and the film electrodes present typical first cyclic voltammetry (CV) curves, which very clearly show one pair of well-defined and symmetrical redox peaks without other redox peaks for the LiFePO_4_ film electrode. The ratio between the anodic and cathodic peak currents is close to 1, implying a good reversibility of lithium insertion and extraction from the LiFePO_4_ film electrode. It should also be noted that the voltage of oxidation peak for the film electrode is more positive than that for the cathode peak, whereas the voltage of reduction peak is almost same. This over-potential phenomenon is in agreement with the Li + insertion/extraction process in an organic electrolyte. It has been reported that peak voltage of anodic process is sometimes more positive due to the decomposition of electrolyte in the organic electrolyte [22]. The appearance of well-defined pair redox peaks shows the redox activity of Fe^2+^/Fe^3+^, which is caused by lithium ion insertion/extraction in LiFePO_4_ during charge and discharge processes. The CV curves indicate that the redox reaction mechanism of LiFePO_4_ in Li_2_SO_4_ aqueous electrolyte is similar to that in organic electrolyte.

**Fig. 4:**
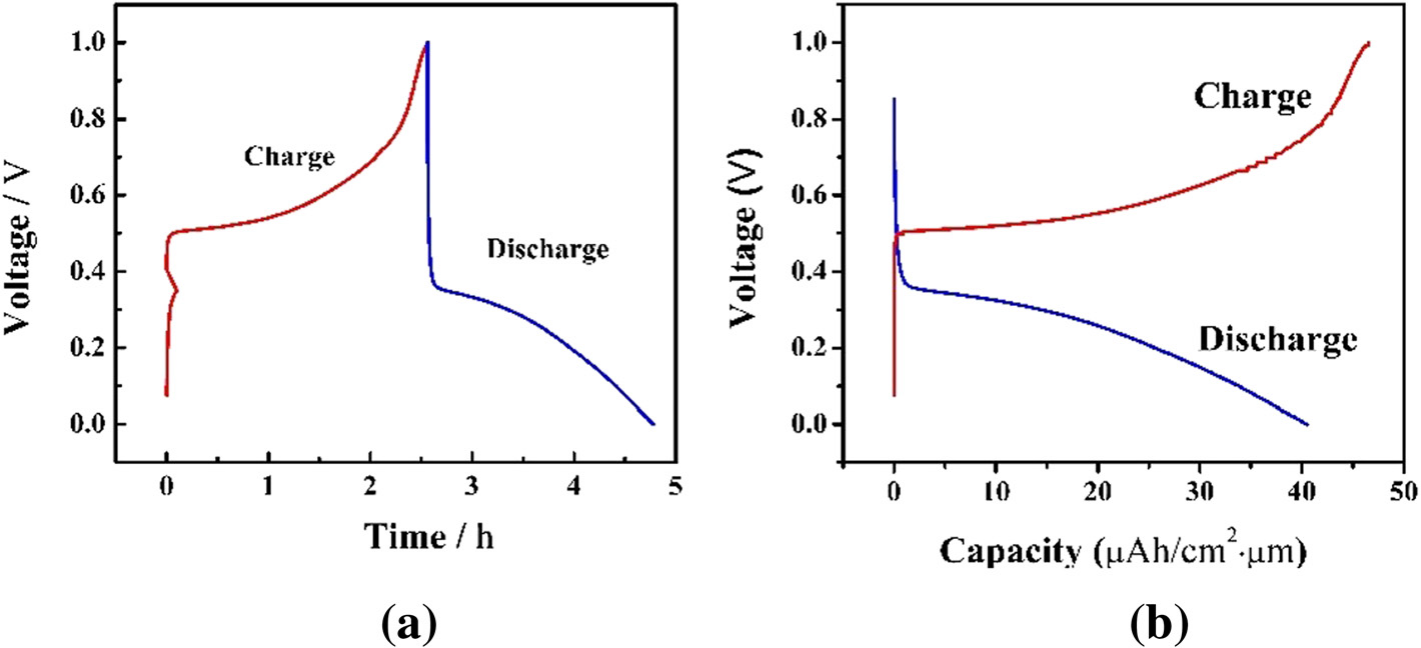
**a** Chronopotentiometry test curves and **b** capacity curves of the LiFePO_4_ thin film in 1 M Li_2_SO_4_ aqueous electrolyte

The galvanostatic charge-discharge curve of the LiFePO_4_ thin film at the constant current density of 10 μAh cm^−2^ from 0 to 1 V during first cycle was shown in Fig. 4. The CP curves in Fig. 4a correspond well to the results of cyclic voltammetry, and CP will further be applied in following EC-AFM characterization. In Fig. 4b, it is obvious that two typical charge and discharge plateaus at 0.5 and 0.3 V appear during the first cycle. The charge-discharge plateaus correspond to the Li^+^ intercalation-deintercalation process in the LiFePO_4_ film electrode, and the corresponding equations of charge-discharge plateaus can be written as two-step reactions:

$$ {\mathrm{FePO}}_4+x{\mathrm{Li}}^{+}+x{\mathrm{e}}^{-}\to x{\mathrm{Li}\mathrm{FePO}}_4+\left(1-x\right){\mathrm{FePO}}_4 $$

$$ {\mathrm{Li}\mathrm{FePO}}_4-x{\mathrm{Li}}^{+}-x{\mathrm{e}}^{-}\to x{\mathrm{FePO}}_4+\left(1-x\right){\mathrm{Li}\mathrm{FePO}}_4 $$

It can also be seen from Fig. 4b that the LiFePO_4_ film delivers an initial discharge capacity of 41 μAh cm^−2^ μm^−1^. As the theoretic density of LiFePO_4_ is 3.6 g cm^−3^, the initial discharge capacity can be calculated to be 113 mAh g^−1^. This result is similar to the findings in previous studies [8].

**Fig. 5:**
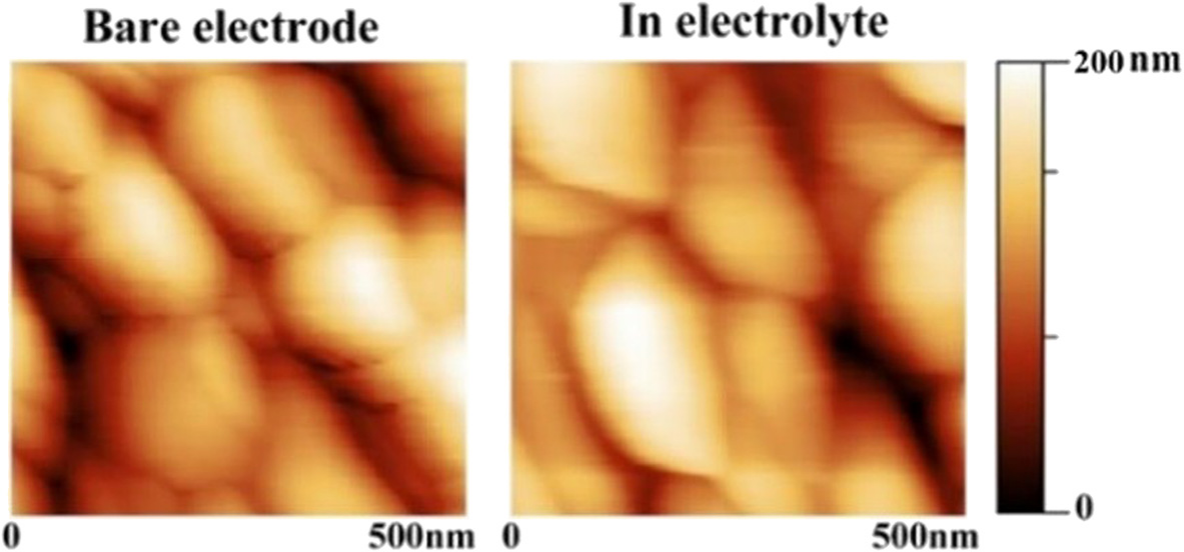
AFM height image of bare and film electrode in electrolyte

Before in situ EC-AFM measurements, representative AFM images of the film electrode in air and in liquid were obtained, as shown in Fig. 5, respectively. From the images, grain boundaries can be clearly seen and no obvious difference between them can be found. The results suggest that the AFM system works very well in the aqueous electrolyte.

**Fig. 6:**
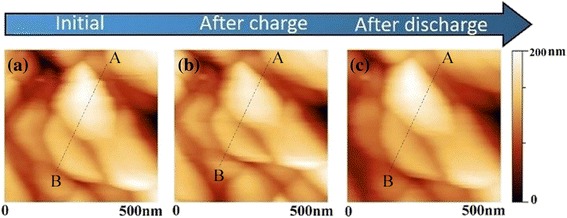
AFM topographic image of the film electrode obtained in aqueous electrolyte **a** at initial state, **b** after charge process, and **c** after discharge process

Figure 6 shows AFM images of the film cathode in aqueous electrolyte, obtained at initial state, after charging and after discharging in CP characterization, respectively. Referring to Fig. 4a, the image in Fig. 6a was taken at the initial state, before CP test. In this case, grains show an average size of ~100 nm, which is consistent with the SEM result in Fig. 2. When the thin film electrode was gradually charged to 1 V at a current of 10 μAh cm^−2^, the CP process was suspended and AFM images were captured, as shown in Fig. 6b. From the image, changes in size and shape of the grains can be obviously observed. More precisely, the size of the grains decrease and the shape became more circular. The image in Fig. 6c was obtained in the end of discharge process in CP. In this case, the size of grains increases and the shape became more similar to that initially observed. To further verify the change of grains, the cross-section line and grain area are quantitative analyzed. Detailed results are shown as follows.

**Fig. 7:**
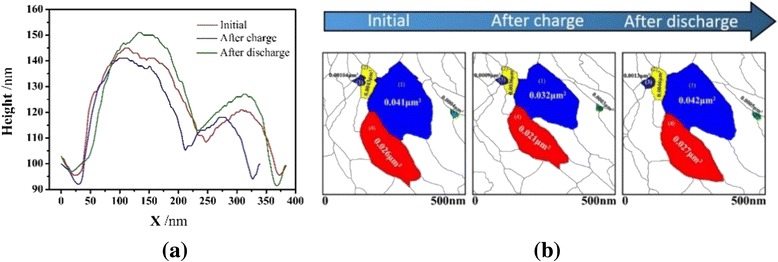
**a** Cross-section profiles of two selected grains and **b** grain area during charge and discharge processes

Figure 7a shows cross-section profiles extracted from the data along the line A–B in Fig. 6, which correspond with the initial state and charge and discharge processes in aqueous electrolyte. It can be seen from the figures that the height of the large grain decreases from 145 to 140 nm during the charge process and increases from 140 to 150 nm during the discharge process, and the small one decreases from 120 to 118 nm during the charge process and increases from 118 to 126 nm during the discharge process, respectively. The area of the grains was also measured and is given in Fig. 7b, which demonstrates, for example, that the area of the large grain (blue color) at initial state is 0.041 μm^2^ and is reduced to 0.032 μm^2^ after charging and that the area increases from 0.032 to 0.042 μm^2^ after discharging process. The other four grains were also measured and compared in the same way as shown in Fig. 7b.

Changes in area and height of more grains numbered in Fig. 6 were also measured in the initial state and during charge and discharge processes, and the results are given in Tables 1 and 2. From the data, it is easily found that the area of the grains decreases after the charge process and increase after the discharge process. Changes in height are consistent with these in area.

**Table 1:** Changes in grain area of the LiFePO_4_ film during initial, charge, and discharge processes

	1	2	3	4	5	6	7	8	9
Initial	0.0195	0.0259	0.04	0.0446	0.0739	0.0813	0.0843	0.11	0.12
After charge	0.0177	0.0236	0.0373	0.0414	0.0686	0.0794	0.0814	0.0993	0.11
After discharge	0.0227	0.0293	0.042	0.0477	0.0771	0.0845	0.091	0.114	0.124

Unit: μm^2^

**Table 2:** Changes in grain height of the LiFePO_4_ film during initial, charge, and discharge processes

	1	2	3	4	5	6	7	8	9
Initial	110	113	118	125	126	128	150	150	163
After charge	103	106	110	116	119	121	145	145	160
After discharge	114	118	123	134	134	135	154	160	168

Unit: nm

Because the sample is mainly composed of LiFePO_4_ phase, as we know, the reason for the decrease and increase of grain area and height are due to phase transformation of the grain between LiFePO_4_ and FePO_4_ during the charge and discharge processes. Li^+^ is extracted from the cathode and LiFePO_4_ changes into FePO_4_ grain during the charge process, while Li^+^ inserts into the cathode and FePO_4_ reversely changes into LiFePO_4_ grain during the discharge process. It has been known that the lattice parameter is different between LiFePO_4_ and FePO_4_ and there is 6.81 % volume change between completely charged and discharged states. Thus, the phase transformation induces the volume change of the grain.

It has been reported that the average grain area decreased from 0.3 to 0.26 μm^2^ during the charge process and increased from 0.26 to 0.33 μm^2^ in the discharge process in nonaqueous electrolyte. The percentage change in grain size is 13.3 % during the charge process and 21.2 % in the discharge process [19]. The results obtained in our present experiments, however, show that the change in average grain size is 19.3 % during charge process and 27.8 % during discharge process, indicating that the volume change in aqueous electrolyte is more serious than that in nonaqueous electrolyte. This phenomenon could be one of the reasons for the faster capacity fading in aqueous electrolyte.

## Conclusions

LiFePO_4_ powder was successfully synthesized by solvothermal method and then thin films were deposited on Au/Si substrate by radio frequency magnetron sputtering. The thin film is composed of LiFePO_4_ phase with olivine structure, and the average grain size is ~100 nm. The cyclic voltammetry shows that both the powder and the thin film have characteristic redox peaks. Charge-discharge measurements at current density of 10 μAh cm^−2^ between 0 and 1 V show that the LiFePO_4_ thin film electrode is able to deliver an initial discharge capacity of 113 mAh g^−1^. With the self-made sample, in situ electrochemical-AFM measurements that are built based on AFM and chronopotentiometry method were conducted. The morphological changes of grains during charge and discharge processes in aqueous electrolyte were directly observed. The change in grain size was analyzed, and the results show that the average size of the grain decreased from 0.067 to 0.062 μm^2^ when it was charged and increased from 0.062 to 0.07 μm^2^ when it was discharged, respectively. This phenomenon is due to the phase transformation between LiFePO_4_ and FePO_4_. In the next stage, we will further study the relationship between the morphology change of grains and capacity loss.

## Abbreviations

ARLB: aqueous rechargeable lithium-ion battery
CP: chronopotentiometry
CV: cyclic voltammetry
EC-AFM: electrochemical atomic force microscopy
